# Encapsulation of the Antioxidant R-(+)-α-Lipoic Acid in Permethylated α- and β-Cyclodextrins: Thermal and X-ray Structural Characterization of the 1:1 Inclusion Complexes

**DOI:** 10.3390/molecules22060866

**Published:** 2017-05-23

**Authors:** Mino R. Caira, Susan A. Bourne, Buntubonke Mzondo

**Affiliations:** Centre for Supramolecular Chemistry Research, Department of Chemistry, University of Cape Town, Rondebosch 7701, South Africa; susan.bourne@uct.ac.za (S.A.B.); buntubonke@gmail.com (B.M.)

**Keywords:** R-(+)-α-lipoic acid, antioxidant, cyclodextrins, thermal analysis, X-ray diffraction, crystal structure, stability

## Abstract

The naturally occurring compound α-lipoic acid (ALA) is implicated in manifold critical biological roles and its potent antioxidant properties and potential for treatment of various diseases have led to its widespread use as a dietary supplement. However, shortcomings of poor aqueous solubility and low thermal stability have hampered its development as a medicinal agent, prompting the use of cyclodextrins (CDs) to address these problems. The paucity of published structural data on the nature of the interactions between ALA and CDs motivated the present study, which describes the synthesis and X-ray structural elucidation of crystalline inclusion complexes between the biologically relevant R-(+)-α-lipoic acid (RALA) and the host molecules permethylated α-CD (TMA) and permethylated β-CD (TMB). Single crystal X-ray diffraction of TMA·RALA·6H_2_O and TMB·RALA revealed significantly different orientations of the RALA molecule within the TMA and TMB cavities, but in both cases the guest molecule is fully encapsulated by the respective parent host molecules and residues of CD molecules of neighboring complex units. While pure RALA melted at 46–48 °C, combined thermal analysis techniques indicated that on heating the respective complexes, the release of RALA occurred at significantly higher onset temperatures, in the range 150–170 °C.

## 1. Introduction

There is considerable current interest in enhancing the performance of compounds with proven medicinal benefits by incorporating them into multi-component systems. In this context, ‘performance’ relates to pharmaceutically relevant properties, primarily bioavailability and chemical stability [1], while the types of multi-component system that have been successfully exploited in enhancing performance include those in which the components are associated via non-covalent interactions. Examples of these constructs are co-crystals formed by linking bioactive molecules to water-soluble biocompatible co-formers via hydrogen bonds [2], and inclusion complexes between bioactive molecules and water-soluble macrocyclic carrier molecules based primarily on hydrophobic interactions [3]. Application of such products has been shown to be beneficial not only for drug delivery, but also for improving drug processability and increasing shelf life. The present study relates to the well-established practice of using cyclodextrins (CDs) to improve the delivery of poorly soluble bioactive compounds [4]. These molecules are cyclic oligosaccharides containing α-1,4-glucopyranose units that are linked by glycosidic bonds. As described in many articles, reviews and monographs, a CD molecule has the shape of a truncated cone with a hydrophilic exterior and a central hydrophobic cavity that can accommodate hydrophobic guest molecules via non-covalent interactions. This enables the bioactive guest molecule to be released intact upon complex dissociation in vivo with a higher concentration than that realized in the absence of the CD. Chemical modification of CDs yields a wide range of derivatives that can thus modulate the release rates of the included bioactive molecules and hence have a significant impact on drug delivery [4]. Since the magnitude of the association constant for a CD–drug complex can vary significantly for a given drug, depending on the structural nature of the CD in question, the rate of drug release will change accordingly. Thus, for example, the maximum drug serum level following oral delivery of a drug using its β-CD complex may be significantly greater than that from its permethylated β-CD (TMB) complex due to the lower value of the complex association constant in the former case.

**Figure 1:**
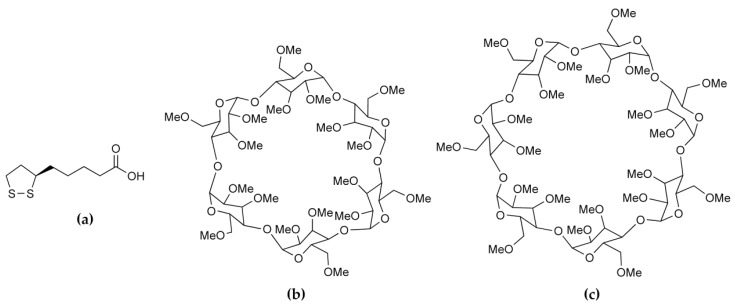
Molecular structures of (**a**) R-(+)-α-lipoic acid (RALA); (**b**) permethylated α-CD (TMA) and (**c**) permethylated β-CD (TMB).

The compound α-lipoic acid (ALA, 1,2-dithiolane-3-pentanoic acid, also known as thioctic acid) is an endogenous fatty acid produced in the mitochondria and it occurs in low concentrations in the human diet (e.g., in various meat products and some fruits and vegetables) [5,6]. With one chiral center, ALA can exist in R- and S-enantiomeric forms, the former, R-(+)-α-lipoic acid (RALA, Figure 1a) being the relevant one in the biological milieu. Following its isolation from the liver in 1951 [7], many studies have been undertaken to determine its biological roles. It is currently known to be intimately involved in the mitochondrial energy metabolism, functioning as an essential cofactor for mitochondrial α-keto acid dehydrogenases. Numerous medicinally beneficial effects of orally-administered ALA have also been identified. The most frequently mentioned is its potent biological antioxidant activity, manifested in its ability to either scavenge various reactive oxygen species (ROS), or alternatively via its induction of other endogenous antioxidants (e.g., ascorbate) [6,8]. However, it is also a metal chelator [6,9] and it has been shown to have antidiabetic [6,10], hypotensive [6,11], anti-aging and anti-inflammatory [6,12] activities, as well as positive effects on neuro-cognitive functions [6,13]. In a recent review, ALA was listed as one of the several nutraceuticals that merit ongoing investigation as “novel promising therapeutics” owing to their potential for treating neuroinflammation and neurodegeneration in Alzheimer’s disease [14].

A drawback in the use of the racemic compound (ALA) as a supplement is its very low aqueous solubility (estimated at 0.003 M at 25 °C in a recent study [15]) and the melting point of 60 °C is relatively low [15]. These physical properties are not pharmaceutically favourable and hence the water-soluble sodium salts (of both ALA and RALA) have been employed in commercially available supplements. However, during the last 20 years, an alternative approach to increasing both the low aqueous solubility and the low thermal stability of ALA has been pursued, namely its complexation with native and modified CDs. The vast majority of such studies have focused on the use of the racemic compound (ALA) rather than RALA due to the relatively higher cost of the latter form, which is in fact less thermally stable than ALA, having a previously reported melting point of 46–49 °C, above which polymerization occurs [16]. Most of the studies conducted with ALA have focused on the solution state and in general, CD inclusion has indeed been shown to increase the aqueous solubility of ALA. However, since the focus of the present study is on RALA and its solid-state inclusion complexes with CDs, pertinent recently published studies treating these aspects are briefly described here. The enhanced stability of RALA by complexation with α-, β- and γ-CD has been demonstrated [16]. Preparation of the solid inclusion complexes and their analyses by differential scanning calorimetry (DSC) and other techniques established that complexed RALA is more stable than untreated RALA when exposed to high temperature and high humidity or acidic pH conditions. Of the three native CDs tested, the host γ-CD was found to confer the highest stability on RALA. Subsequently, a report based on a comprehensive study by microscopy and spectroscopy of single crystals of the 3:2 α-CD·RALA inclusion complex appeared [17]. Channel-type complexation was deduced from the close similarity of its powder X-ray diffraction (PXRD) pattern to that of known isostructural complexes. Using the combined results from several techniques, it was determined that the mode of RALA inclusion involved almost complete enclosure of the dithiolane ring within the host cavity, this residue being oriented almost perpendicular to the host ring-plane. With a 3:2 host–guest stoichiometry, four possible topological structures for the crystalline complex were proposed. More detailed structural data were reported for a CD·RALA inclusion complex [18], namely β-CD·RALA, following its crystallographic analysis based on laboratory PXRD data [19]. This involved model building and rigid-body Rietveld refinement with stereochemical restraints leading to a structural model comprising non-H atoms only, in which the guest molecule was reported to be located near the primary face of the β-CD molecule, with the side chain in a folded conformation such that the terminal –COOH group protrudes from the secondary side of the host, occupying an interstitial site. A search for X-ray crystal structures of CD–ALA inclusion complexes in the Cambridge Structural Database [20] revealed only the former model involving specifically the R-enantiomer (RALA) as a guest.

Given the current level of interest in exploiting RALA as a potential drug to treat a wide array of medical conditions, preferred modes of interaction between RALA and receptor mimics, such as CDs, are highly relevant. Thus, the present study was undertaken to address the paucity of structural information available on such CD–RALA interactions. To this end, the permethylated CDs selected as host compounds were hexakis (2,3,6-tri-*O*-methyl)-α-CD (TMA, Figure 1b) and heptakis(2,3,6-tri-*O*-methyl)-β-CD (TMB, Figure 1c). Methylated CDs were chosen specifically since they are less prone than native CDs to yield inclusion complexes that display guest disorder. Since only one distinct guest molecule is involved, possible disorder that can occur when both enantiomers are included in a CD is eliminated. Recent studies with such CDs in our laboratory have revealed new insights into the modes of guest inclusion and unusual complex crystal packing arrangements that are realized with other notable bioactive guest molecules such as the antioxidant resveratrol [21] and the anticancer agent 2-methoxyestradiol [22]. Furthermore, it was of interest to determine the extent to which the thermal stability of RALA might be modified by its inclusion in the selected host molecules. The strategies for achieving the two objectives above were the use of single crystal X-ray diffraction to elucidate structural details of CD·RALA inclusion complexes and thermal analysis techniques to investigate the effect of CD inclusion on the thermal stability of RALA. As detailed below, the crystallization of the inclusion complexes TMA·RALA·6H_2_O **1** and TMB·RALA **2** was successful and their structural elucidation by single crystal XRD revealed distinct differences in the orientations of the RALA molecule in the respective host cavities, guest conformational differences and the significant role that included water molecules in complex **1** play in guest stabilization and crystal packing. It is emphasised that there is no intention in this report to motivate the present study on the basis of delivery of RALA through the use of cyclodextrins, since to our knowledge TMA and TMB are not used in that context. However, they meet the requirements of the present solid-state investigation very well.

## 2. Results and Discussion

### 2.1. Single Crystal X-ray Diffraction

The high quality of the complex crystals afforded excellent diffraction data sets, enabling location of all hydrogen atoms (including, remarkably, those on the water molecules in complex **1**). Further evidence of the high accuracy and precision of the analyses is reflected in the exceptionally low residual indices for complexes of this type. Relevant crystallographic data and refinement details for the two inclusion complexes are as follows.

Crystal data for TMA·RALA·6H_2_O **1**, [C_54_H_96_O_30_·C_8_H_14_O_2_S_2_·6H_2_O] (M = 1539.71 g/mol): orthorhombic, space group P2_1_2_1_2_1_ (no. 19), a = 15.1893(7) Å, b = 21.7433(11) Å, c = 23.4588(11) Å, V = 7747.6(6) Å^3^, Z = 4, T = 173(2) K, μ(MoKα) = 0.159 mm^−1^, D_calc_ = 1.320 g/cm^3^, 72,052 reflections measured (3.2° ≤ 2Θ ≤ 50.5°), 19,280 unique (R_int_ = 0.0423) which were used in all calculations. The final values of R_1_ and wR_2_ were 0.0465 [I > 2σ(I)] and 0.1170 respectively (all data).

Crystal data for TMB·RALA **2**, [C_63_H_112_O_35_·C_8_H_14_O_2_S_2_] (M = 1635.83 g/mol): orthorhombic, space group P2_1_2_1_2_1_ (no. 19), a = 15.1076(5) Å, b = 21.1330(8) Å, c = 27.3002(10) Å, V = 8716.1(5) Å^3^, Z = 4, T = 173(2) K, μ(MoKα) = 0.145 mm^−1^, D_calc_ = 1.247 g/cm^3^, 102,702 reflections measured (3.6° ≤ 2Θ ≤ 56.6°), 21,659 unique (R_int_ = 0.0430) which were used in all calculations. The final R_1_ was 0.0431 [I > 2σ(I)] and wR_2_ was 0.1079 (all data).

**Figure 2:**
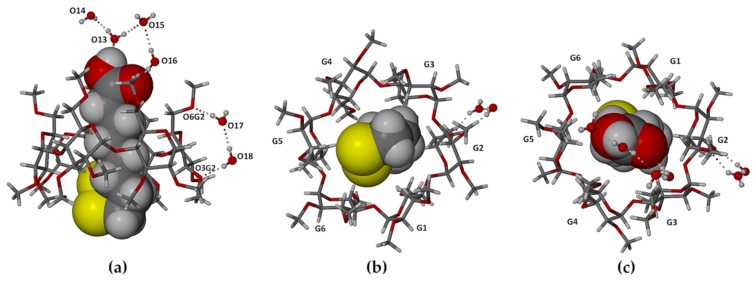
Complex **1** with its complement of water molecules: (**a**) a view showing the dithiolane ring of RALA located at the secondary side of the host and the carboxylic acid group at the primary side; (**b**) a view from the host secondary side; and (**c**) a view from the primary side. The symbols Gn designate the six methylated glucopyranose rings. Unless otherwise stated, standard colors are employed for atoms (H: white, C: grey, O: red, S: yellow).

The asymmetric unit of complex **1** (Figure 2) comprises a 1.1 TMA·RALA unit and six water molecules. Encapsulation of the RALA molecule occurs with its side chain in a fully extended conformation, the bulkier dithiolane ring moiety adopting an envelope conformation (flap at S2) and being located at the secondary (wider) side of the host molecule and the carboxylic acid group at the primary side. With the six methoxymethyl substituents at the TMA primary rim also fully extended, the RALA molecule is completely sheathed within the hydrophobic cavity environment. However, the hydrophilic –COOH group is stabilized via hydrogen bonding with water molecules (oxygen atoms O13, O16), acting both as the donor and acceptor (Figure 2a). Similar hydrogen bonding patterns involving phenolic groups of the antioxidant resveratrol and water molecules were observed in its inclusion complexes with TMA and DMB (dimethylated β-CD) [21]. The secondary side of the complex unit is instead in contact with methoxyl groups of three neighboring TMA molecules. The mode of RALA inclusion observed here thus epitomizes its amphiphilic nature, manifested in its ability to act as an antioxidant in different biological microenvironments (e.g., cytosol, plasma membranes, serum, lipoproteins) [23,24]. Figure 2b,c show the terminal groups of the RALA molecule and their environments viewed from the (wider) secondary and (narrower) primary sides of the TMA molecule respectively.

**Figure 3:**
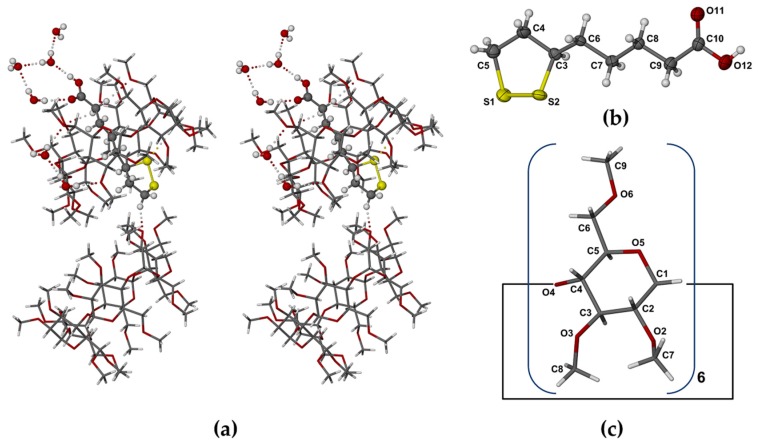
(**a**) Stereoscopic view of principal host–guest hydrogen bonds and those involving peripheral water molecules; (**b**) the conformation and numbering of the included RALA molecule with thermal ellipsoids of the non-H atoms drawn at the 50% probability level; (**c**) the structure of TMA with atomic nomenclature of the representative methylglucopyranose unit.

The location of the –COOH group at the primary side of the TMA molecule is stabilized not only by the H-bonds involving extra-cavity water molecules, but also by two (guest methylene) C-H···O6(primary methoxyl) H-bonds (Figure 3a). The extended conformation of the RALA molecule is associated with complementary tethering of the dithiolane ring at the host secondary side, effected by a (host methine)C–H···S H-bond and an intermolecular (guest)C–H···O3(host) H-bond whose acceptor atom belongs to a 2_1_-related TMA molecule in a neighboring complex unit. Hydrogen bond data are listed in the Supplementary Materials (Table S1). These include stabilizing hydrogen bonds of the following types: intramolecular and intermolecular host(C–H)···O(host), water(O–H)···O(host methoxyl) and O–H···O between water molecules. Guest and host numbering schemes are shown in Figure 3b,c.

Parameters describing the observed conformations of CD molecules are frequently based on deviations from their ideal ‘undistorted’ structures, using the least-squares plane through the glycosidic (O4) oxygen atoms as a primary reference feature. An analysis of the geometrical parameters describing the host TMA molecule in its complex with RALA (Supplementary Materials, Table S2) shows that the six glycosidic O4 atoms form a fairly regular hexagon, with O4···O4′···O4′′ angles (parameter a) in the range 116.7°–122.7°, and that they are coplanar to within ~0.08 Å (parameter α). However, there is a wide range in the values of the tilt angle (τ), which measures the extent to which each glucopyranose ring has its primary side tipped towards the cavity (+) or away (−) from it. Here, inclusion of the RALA molecule in TMA results in a specific, unique pattern of alternating small and large positive values of τ, with overall range 9.8°–22.7°. These geometrical parameters differ significantly from those found, for example, in the TMA resveratrol inclusion complex (respective ranges for two crystallographically-independent host molecules are 3.2°–6.4° and 5.7°–10.3°). This confirms our previous observations that the topological features of the host cavity (reflected in these parameters) are able to adjust to achieve optimum interaction with a particular guest molecule and its precise conformation and mode of inclusion. This phenomenon has been cited as evidence of ‘mutual induced fit’ [21,22,25,26,27], in cases involving relatively small host and guest molecules rather than macromolecules.

**Figure 4:**
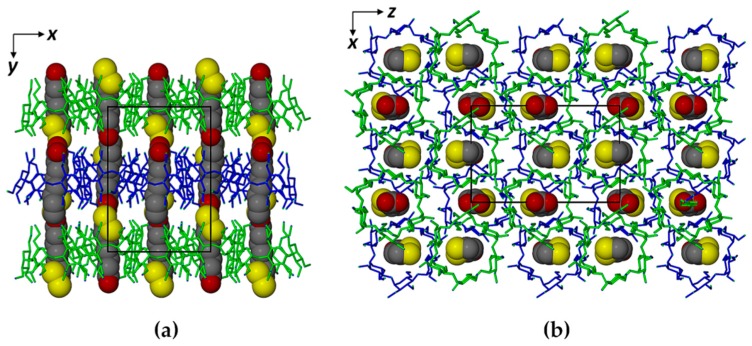
(**a**) The [001] projection and (**b**) the [010] projection of the TMA·RALA·6H_2_O structure.

The absence of isostructural TMA complexes in the Cambridge Structural Database necessitated the preparation of the crystal structure solution by ab initio direct methods and the packing arrangement in the crystal of hydrated TMA·RALA is thus a new structure. As shown in Figure 4a, a major structural feature is the formation of discrete layers of complex units at intervals of b/2, related by the two-fold screw axis parallel to b and indicated by the alternating green and blue bands of TMA molecules. This feature is responsible for the reflection from the (020) planes being the dominant feature in the PXRD pattern [28] (Supplementary Materials, Figure S1). However, in Figure 4b, it is evident that this structure is not of the ‘channel-type’, since each CD complex unit in one layer is located directly above an interstice at the junction of three complex units in the layer below.

The TMA molecules are drawn in stick representation and the RALA molecules in space-filling mode. Water molecules and H atoms are omitted for clarity.

**Figure 5:**
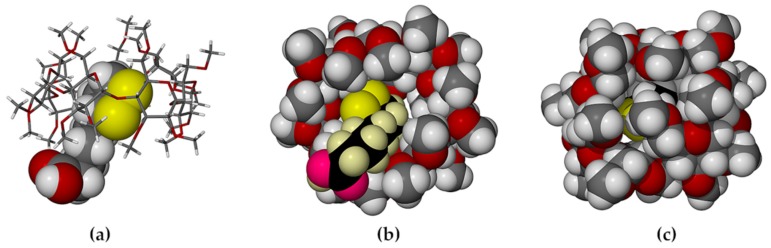
Partial inclusion of RALA in the host TMB, (**a**) side-view; (**b**) view from the secondary side of the host; (**c**) view from the primary side of the host. In (**b**, **c**), a different guest color scheme from that in (**a**) is employed in order to distinguish it from the host atoms (specifically, H: cream, C: black, O: magenta).

In the anhydrous complex **2** (TMB·RALA), the host contains seven methylglucose units (as opposed to six for TMA). The mode of RALA inclusion in TMB is shown as a general view in Figure 5a. Methoxymethyl groups on the primary side of TMB are folded towards the center of the cavity and their steric bulk acts as a ‘lid’, preventing deep guest penetration. This is frequently observed in TMB complexes [20]. Effectively, the dithiolane ring (in a twist conformation, twist on C4–C5) and the methylene group, attached to C3 (Figure 3b), are encapsulated by the host molecule while the remaining portion of the side chain [–(CH_2_)_3_–COOH] protrudes from the host’s secondary side. Figure 5b,c show views of the complex unit from the host secondary and primary sides respectively; the latter clearly reflecting efficient blocking of the primary side by the methoxymethyl groups. As described below, the side chain of the RALA molecule is not fully extended and the carboxylic acid group is thus able to engage in intermolecular hydrogen bonding, as described below.

**Figure 6:**
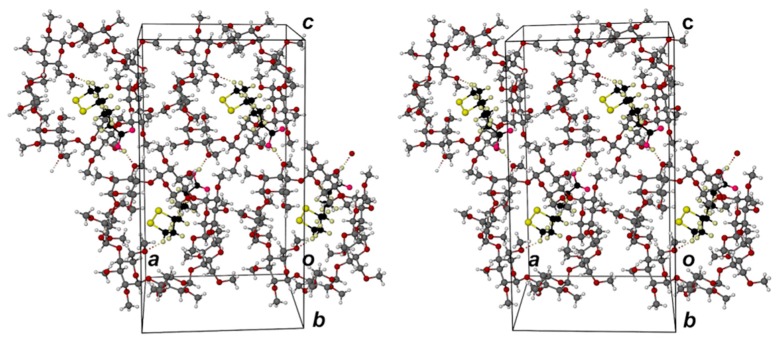
Stereoview of the array of TMB·RALA complex units related by a 2_1_-axis parallel to the crystal axis *a*, showing the associated hydrogen bonding interactions.

The host–guest interactions are primarily hydrophobic. A single (guest) C–H···O6(host) H-bond contributes to the stabilization of the dithiolane ring location in the TMB cavity (Figure 6), while the –COOH group is a donor in a hydrogen bond to a O3G2’ atom of a neighboring TMB molecule related by the two-fold screw-axis parallel to the crystal *a*-axis. These are the only significant H-bonds in which the RALA molecule engages. However, their combination leads to an infinite array of connected complex units along the *x*-direction as shown in Figure 6. Hydrogen bond data are listed in the Supplementary Material (Table S3).

**Figure 7:**
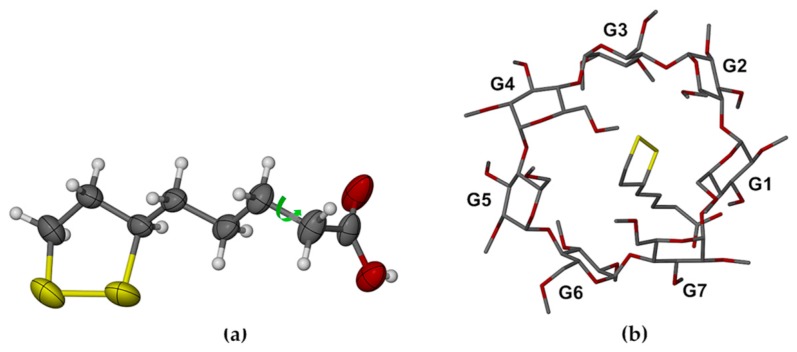
(**a**) The conformation of the RALA molecule in its complex with TMB, with the dihedral angle C7–C8–C9–C10 highlighted and the atoms drawn as thermal ellipsoids at the 50% probability level, and (**b**) view of the TMB·RALA complex from the secondary side of the host molecule and normal to the least-squares plane through the O4-heptagon, revealing the extreme variations in the tilt angles of the seven methylated glucopyranose rings.

Whereas the RALA molecule in complex **1** was fully encapsulated within the TMA host molecule with its side chain in the extended conformation, that in complex **2** had its side chain extended only from C6 through C9, with the –CH_2_-COOH moiety rotated around the C8–C9 bond (torsion angle C7–C8–C9–C10 = 65.7(5)°), this conformation being associated with the establishment of the stabilizing hydrogen bond –COOH···O3G2’ (Figure 6 and Figure 7a).

Comparison of Figure 3b and Figure 7a also revealed the lower average thermal motion of the atoms C6 through O12 comprising the RALA side chain in the TMA complex (U_eq_ range 0.030–0.062 Å^2^), which was consistent with its tight confinement in the cavity. Instead, the major portion of the RALA side chain in TMB·RALA protruded from the cavity, reflected in the significantly higher U_eq_ range of 0.054–0.093 Å^2^ at the same temperature. The dithiolane ring of the RALA molecule rested on one side of the TMB cavity wall (Figure 6) while the apparent void on the other side was inaccessible due to close contacts with primary methoxyl groups of another host molecule (not shown). These features resulted in the TMB molecule being severely distorted (Figure 6 and Figure 7b), as evidenced by several of the geometrical parameters (Supplementary Materials, Table S4), especially the observed methylglucose ring tilt angles, which span a very wide range, from −13.0° (for G6) to 49.3° (for G4). The overall packing arrangement of the TMB molecules in the complex crystal belongs to a fairly common herring-bone type that characterizes a sizeable series of isostructural complex crystals [20]. The computed PXRD pattern for the TMB–RALA crystal (Supplementary Materials, Figure S2) serves as a useful reference for assessing the purity of bulk samples of the inclusion complex. The crystal structure is stabilized primarily by a large number of intramolecular and intermolecular host(C–H)···O(host) hydrogen bonds.

In both inclusion complexes, each molecule of RALA is completely isolated, being surrounded by CD molecules only and thus the RALA molecules are protected from interacting with one another.

### 2.2. Thermal Analysis of the Starting Materials and the CD Inclusion Complexes

**Figure 8:**
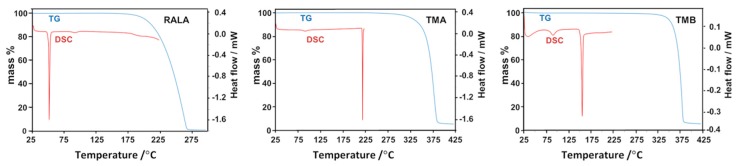
Combined thermogravimetric (TG) and differential scanning calorimetric (DSC) traces for RALA, TMA and TMB.

Figure 8 shows the thermogravimetric (TG) and differential scanning calorimetric (DSC) curves for RALA, TMA and TMB. The principal feature of pure RALA is its low melting point (onset 47 °C, peak 50 °C in the trace of Figure 8), above which it is known to undergo facile polymerization; this thermal instability hampers its utility in industrial applications [16]. In contrast, the two CDs employed in the study were thermally much more stable, TMA melting at 219 °C and TMB at 155 °C.

**Figure 9:**
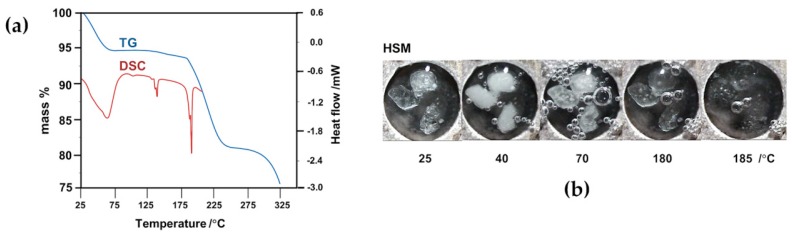
(**a**) Combined TG and DSC traces with (**b**) complementary hot stage microscopy (HSM) micrographs for the complex TMA·RALA·6H_2_O.

In order to determine the extent to which the inclusion of RALA in the chemically modified CDs described above might alter the thermal stability of the pure guest (melting range 47–54 °C, determined in our experiments), crystals of the complexes **1** and **2** were subjected to thermogravimetry, differential scanning calorimetry and hot stage microscopy (HSM), representative results of which are shown in Figure 9. The combination of these methods also enabled calculation of the complex stoichiometries, as detailed below. Heating complex **1** between 25 °C and 80 °C (Figure 9a) led to crystal dehydration, indicated by the discrete initial TG mass loss of 5.9 ± 0.1%, corroborated by the broad, major DSC endotherm as well as the occurrence of copious bubbling observed from the crystals immersed in silicone oil (Figure 9b). Assuming a TMA:RALA molar ratio of 1:1, this mass loss would correspond to five water molecules per hydrated complex unit (calcd. value 5.9%). A subsequent small, gradual mass loss of 1.1 ± 0.1% between 120 °C and 170 °C accompanied by a small endotherm in DSC at ~140 °C interpreted as further dehydration, accounted for the loss of an additional water molecule (calcd. value 1.2%). The endothermic peaks corresponding to dehydration were generally composite, indicating partly overlapping, stepwise loss of water molecules, a notion that could be reconciled with the crystal structure analysis which revealed water molecules in a variety of environments, engaging in hydrogen bonding to various extents.

Also noteworthy is the TG estimate of six water molecules per TMA·RALA unit, which is in accord with the number of water molecules located in the X-ray structural analysis, none of them showing disorder. Melting of the anhydrous crystal followed at approximately 185 °C, the temperature at which release of RALA commenced, with the complete process involving a discrete mass loss of 13.4 ± 0.1% between 170 °C and 270 °C, corresponding to the loss of one molecule of RALA from the dehydrated complex (calcd. 13.4% for 1:1 host–guest stoichiometry). This major mass loss was accompanied by a large, sharp endotherm in DSC. The principal finding from these experiments is that inclusion of RALA in the host TMA results in very significant thermal stabilization of the guest, which is released from the complex crystal at ~185 °C.

**Figure 10:**
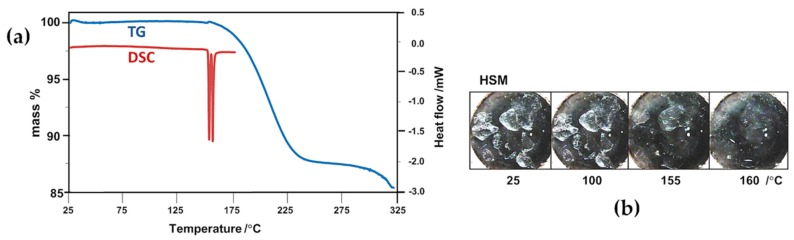
(**a**) Combined TG and DSC traces with (**b**) complementary HSM micrographs for the complex TMB·RALA.

Thermal analysis of complex **2** (Figure 10) was less complicated since there was no evidence of crystal dehydration from any of the methods employed. Instead, two extremely sharp endotherms appeared, peaking at ~154 °C and ~157 °C. The first was interpreted as being due to the commencement of dissociation of the TMB complex of RALA, corroborated by the initiation of mass loss in TG. The second endotherm, following almost immediately, was attributed to the fusion of the residual TMB, one of whose polymorphs is known to melt at ~157 °C [29]. The TG mass loss in the temperature range 150 °C–265 °C was determined as 12.7 ± 0.1%, which corresponds to one molecule of RALA per TMA complex unit (calcd. 12.6% for 1:1 host–guest stoichiometry). These results are in accord with the X-ray structure determination which confirmed the absence of water in the complex crystals and revealed only one (ordered) guest molecule per host molecule. Apart from an indication of possible melting of residual TMB at around 155 °C, the HSM micrographs did not reveal any significant thermal events. Importantly, the guest RALA is thermally stabilized to a very significant extent by inclusion in the host TMB, being released at a temperature that far exceeds its own melting point of 46–48 °C. In summary, the process of inclusion of RALA in both TMA and TMB, which isolates RALA molecules from one another in the solid state, eliminates low-melting behaviour and circumvents the polymerization that occurs upon heating crystals of pure RALA at temperatures above its melting point [16].

## 3. Materials and Methods

TMA and TMB were acquired from Cyclolab Ltd., (Budapest, Hungary) and enantiomerically pure R-(+)-α-lipoic acid (RALA) was purchased from Sigma-Aldrich (St. Louis, MO, USA). Single crystals of the two inclusion complexes were prepared by the co-precipitation method. In each case, equal amounts (0.014 mmol) of the methylated CD and RALA were dissolved in 2 mL Milli-Q^®^ water at 5 °C. Rapid stirring of this mixture for 1 h followed. In order to achieve efficient dissolution of the guest and its complexation with the host, temperature cycling was employed, i.e., heating the mixture to ~40 °C, stirring rapidly for 1 h and subsequently cooling, and repeating this procedure several times. The final cycle involved stirring for 2 h at 5 °C and then filtering the resulting solution into a clean, sealed vial and allowing crystallization to occur at 40 °C. Colorless single crystals appeared after two days.

Intensity data-collections were performed on a Bruker Apex II four-circle diffractometer (Madison, WI, USA) with MoKα X-rays and with the crystal specimens bathed in a constant stream of nitrogen vapor at 173(2) K. The TMA·RALA·6H_2_O complex structure was solved using ab initio direct methods since no isostructural TMA complex data were available in the CSD [20]. Isomorphous replacement using host atomic co-ordinates from an isostructural complex (CSD refcode BIFGUT) was used to develop the structural model of the TMB complex. Full technical details of X-ray data-collections, data reduction, structure solutions, least-squares refinements and computer programs employed are listed in the CIF files (Supplementary Material).

HSM images were viewed using a Nikon SMZ-10 stereomicroscope (Tokyo, Japan) and micrographs were recorded using a Linkam THMS600 hot stage with temperature controlled by a Linkam TP92 heating unit. Crystals were immersed in silicone oil during the heating program. Thermogravimetry was performed using a TA Q500 Thermogravimetric Analyzer (TA Instruments, New Castle, DE, USA) with samples in the 1.5–3.5 mg mass range placed in alumina crucibles and heated at a rate of 10 K min^−1^ over the temperature ranges 25–300 °C (RALA), 25–425 °C (TMA, TMB) and 25–325 °C (complexes **1** and **2**). A nitrogen gas purge with flow rate 60 mL min^−1^ was used. Data were processed with TA Instruments Universal V4.7A. DSC traces were recorded on TA Q200 and TA Q20 instruments (TA Instruments, New Castle, DE, USA), using a heating rate of 10 K min^−1^ with samples in the mass range 1.5–3.5 mg placed in vented platinum pans, and a nitrogen purge rate of 50–60 mL min^−1^. Temperature ranges were 25–225 °C for the pure components RALA, TMA and TMB, 25–210 °C for complex **1**, and 25–180 °C for complex **2**. The ranges for the inclusion complexes were selected to avoid possible damage to the DSC thermocouple that can occur with sample spillage due to foaming at higher temperatures. Data were processed with TA Instruments Universal V4.7A. TGA and DSC measurements for the complexes were generally recorded in triplicate, enabling accurate complex host–guest stoichiometries to be determined.

## 4. Conclusions

Analyses of the crystal structures of the inclusion complexes TMA·RALA·6H_2_O and TMB·RALA have provided the most accurate and detailed information to date on the molecular interactions that bind the prominent antioxidant molecule, RALA, to two well-known chemically modified cyclodextrin host molecules. This information, which includes the accurate structural model of the RALA molecule, has potential use in the computation and prediction of possible modes of interaction of RALA with biological receptors in its envisaged further development as a medicinal agent. The biological relevance of the R-enantiomer of α-lipoic acid prompted its use in the preparation of the complexes; this choice also eliminated possible complications that might have arisen with the use of the racemic compound (viz. concomitant diastereomeric complex formation). Despite the finding that the modes of RALA inclusion are significantly different in the host molecules TMA and TMB, partly owing to their generally preferred cavity topologies, the amphiphilic nature of the RALA molecule was clearly evident from the hydrophobic and hydrophilic crystalline environments in which the respective residues of RALA, the dithiolane ring and the carboxylic acid group, are situated. The combination of thermoanalytical techniques employed was invaluable, serving the dual purpose of establishing accurate complex stoichiometries and assessing the remarkable thermal stabilization of RALA effected by its inclusion in the host molecules TMA and TMB.

## Supplementary Materials

The following are available online at: Tables S1–S4, Figures S1 and S2, CIF files. CCDC 1524720 and 1524721 contain the supplementary crystallographic data for this paper. These data can be obtained free of charge via, http://www.ccdc.cam.ac.uk/conts/retrieving.html (or from the CCDC, 12 Union Road, Cambridge CB2 1EZ, UK; Fax: +44-1223-336033; E-mail: deposit@ccdc.cam.ac.uk).
