# Supplementary material for: Encapsulation of the Antioxidant R-(+)-α-Lipoic Acid in Permethylated α- and β-Cyclodextrins: Thermal and X-ray Structural Characterization of the 1:1 Inclusion Complexes
Source: Molecules. 2017 May 23;22(6):866. doi: 10.3390/molecules22060866 (PMC6152681; doi:10.3390/molecules22060866)
Supplement: Supplementary file 1 [file molecules-22-00866-s001.zip › Supplementary Material/SUPPLEMENTARY DATA.docx]

**SUPPLEMENTARY MATERIAL FOR THE PAPER**

Encapsulation of the antioxidant R-(+)-α-lipoic acid in permethylated α- and β-cyclodextrins: thermal and X-ray structural characterization of the 1:1 inclusion complexes

Mino R Caira, Susan A Bourne and Buntubonke Mzondo,

Centre for Supramolecular Chemistry Research, Department of Chemistry, University of Cape Town, Rondebosch 7701, South Africa.

**TABLE S1: Hydrogen bond data for complex 1: TMA⋅RALA⋅6H_2_O**

Hydrogen bonds with H⋅⋅⋅A < r(A) + 2.000 Angstroms and <DHA > 110 deg.

**D-H d(D-H) d(H⋅⋅⋅A) <DHA d(D⋅⋅⋅A) A**

C5-H5A 0.990 2.566 142.10 3.402 O3G1 [ x+1/2, -y+1/2, -z+1 ]

C9-H9A 0.990 2.577 169.01 3.554 O6G6

C9-H9B 0.990 2.650 137.33 3.443 O6G4

C6G1-H61A 0.990 2.427 148.10 3.309 O5G2

C6G2-H62A 0.990 2.561 139.75 3.378 O5G3

C7G2-H72A 0.980 2.621 136.32 3.398 O5G6 [ -x+1/2, -y+1, z-1/2 ]

C8G2-H82B 0.980 2.436 122.49 3.075 O2G2

C9G2-H92C 0.980 2.643 150.09 3.527 O16

C2G3-H2G3 1.000 2.403 155.62 3.339 O5G5 [ -x+3/2, -y+1, z-1/2 ]

C6G3-H63A 0.990 2.506 129.89 3.232 O5G4

C6G4-H64B 0.990 2.425 151.52 3.329 O5G5

C7G4-H74A 0.980 2.563 128.87 3.268 O3G3

C8G4-H16 0.980 2.606 111.86 3.107 O2G5

C9G4-H94C 0.980 2.621 138.15 3.414 O652_b

C2G5-H2G5 1.000 2.540 140.53 3.373 O5G3 [ -x+3/2, -y+1, z+1/2 ]

C6G5-H6G2_a 0.990 2.550 138.66 3.357 O5G6

C7G5-H75A 0.980 2.644 144.38 3.488 O5G3 [ -x+3/2, -y+1, z+1/2 ]

C2G6-H2G6 1.000 2.603 168.06 3.587 O5G2 [ -x+1/2, -y+1, z+1/2 ]

C3G6-H3G6 1.000 2.859 159.49 3.812 S2

C6G6-H66B 0.990 2.501 147.09 3.375 O5G1

C6G6-H66B 0.990 2.600 145.00 3.458 O6G1

O12-H12 0.840 1.809 161.05 2.618 O13

O13-H13A 0.820 2.039 168.46 2.847 O14

O16-H16A 0.820 2.117 173.85 2.934 O11

O18-H18B 0.820 2.068 171.76 2.882 O3G2

O15-H15B 0.820 2.217 155.79 2.984 O6G1 [ x+1/2, -y+3/2, -z+1 ]

O18-H18A 0.820 1.934 172.61 2.749 O17

O17-H17A 0.820 2.113 149.88 2.853 O3G4 [ -x+3/2, -y+1, z-1/2 ]

O16-H16B 0.820 2.092 170.13 2.904 O15

O15-H15A 0.820 1.950 166.48 2.754 O18 [ -x+1, y+1/2, -z+1/2 ]

O17-H17B 0.820 2.117 150.11 2.857 O6G2

O14-H14A 0.820 2.479 121.39 2.989 O3G5 [ -x+1, y+1/2, -z+3/2 ]

O14-H14A 0.820 2.385 152.50 3.136 O2G6 [ -x+1, y+1/2, -z+3/2 ]

O14-H14B 0.820 2.105 171.23 2.918 O6G3 [ x-1/2, -y+3/2, -z+1 ]

O13-H13B 0.820 2.017 164.99 2.817 O15

**TABLE S2. Geometrical parameters^**^for the host molecule in the crystal**

**of complex 1 (TMA⋅RALA⋅6H_2_O)**

| **Residue** | ***r*  (Å)** | ***D* (Å)** | ***a* (°)** | ***d* (°)** | ***α*^a^ (Å)** | ***D_3_*^b^ (Å)** | ***τ^c^* (°)** |
| --- | --- | --- | --- | --- | --- | --- | --- |
| G1 | 4.227 | 4.153 | 121.9 | 2.7 | 0.028 | 3.302 | 9.8 |
| G2 | 4.373 | 4.426 | 118.0 | -4.3 | -0.011 | 3.211 | 21.1 |
| G3 | 4.308 | 4.176 | 119.5 | 5.8 | 0.030 | 3.377 | 12.3 |
| G4 | 4.209 | 4.461 | 122.7 | -5.6 | -0.063 | 3.338 | 22.7 |
| G5 | 4.404 | 4.135 | 116.7 | 4.4 | 0.079 | 3.276 | 11.5 |
| G6 | 4.279 | 4.455 | 120.9 | -2.6 | -0.063 | 3.144 | 19.4 |

Mean e.s.d.s: **^a^**0.002 Å; **^b^**0.004 Å; **^c^**0.1°

^**^The listed parameters are defined as follows:

***r***, the distance of each O4 atom from the centroid of the O4-polygon;

***D***, the glycosidic O4⋅⋅⋅O4′ distance;

***a***, the O4⋅⋅⋅O4′⋅⋅⋅O4′′ angle;

***d***, the O4⋅⋅⋅O4′⋅⋅⋅O4′′⋅⋅⋅O4′′′ torsion angle;

***φ***, the intersaccharidic angle C1(n+1)-O4(n)-C4(n);

***D_3_***, the O2⋅⋅⋅O3′ intra-ring distance;

***α***, the deviation of each O4 atom from the mean O4-plane;

***τ***_,_ tilt angle: the angle between the plane containing the atoms O4, C4, C1 and O4′ of a given glucose ring and the mean O4-plane.

**FIGURE S1. Calculated PXRD pattern for complex 1 (TMA⋅RALA⋅6H_2_O)**

(PXRD pattern based on refined single crystal X-ray data at 173(2) K and computed

with program Lazy Pulverix [1] for CuKα_1_ radiation)


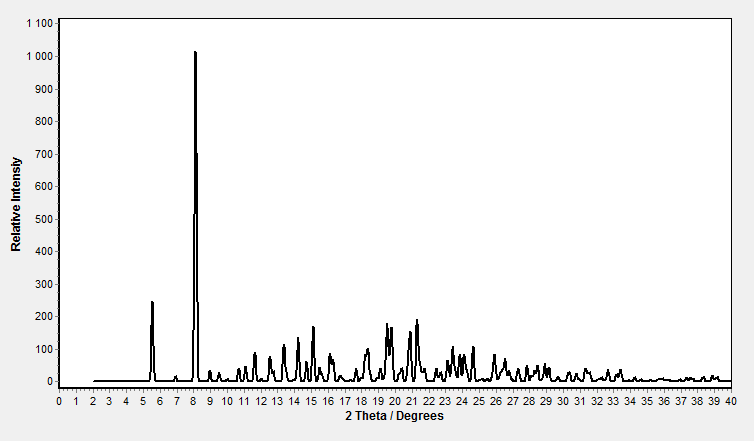


**Relative intensity**

[1]. K. Yvon, W. Jeitschko and E. J. Parthé, *J. Appl. Crystallogr.,* 1977, **10**, 73– 74.

**TABLE S3: Hydrogen bond data for complex 2: TMB⋅RALA**

Hydrogen bonds with H⋅⋅⋅A < r(A) + 2.000 Angstroms and <DHA > 110 deg.

**D-H d(D-H) d(H⋅⋅⋅A) <DHA d(D⋅⋅⋅A) A**

O12-H12 0.840 1.941 160.30 2.747 O3G2 [ x-1/2, -y+1/2, -z+1 ]

C5-H5B 0.990 2.624 132.05 3.368 O6G4

C1G1-H1G1 1.000 2.500 170.46 3.490 O6G6 [ -x+3/2, -y+1, z+1/2 ]

C2G1-H2G1 1.000 2.542 143.45 3.398 O5G5 [ -x+3/2, -y+1, z+1/2 ]

C2G1-H2G1 1.000 2.579 148.85 3.474 O6G5 [ -x+3/2, -y+1, z+1/2 ]

C6G1-H6G2 0.990 2.591 121.47 3.221 O5G7

C6G2-H6G3 0.990 2.440 154.02 3.358 O5G1

C7G2-H7G5 0.980 2.636 162.45 3.582 O2G1 [ x+1/2, -y+1/2, -z+1 ]

C8G2-H8G4 0.980 2.573 110.87 3.062 O2G1

C1G3-H1G3 1.000 2.390 125.09 3.076 O3G4

C4G3-H4G3 1.000 2.485 154.29 3.413 O3G7 [ x+1, y, z ]

C6G3-H9 0.990 2.344 134.02 3.115 O5G2

C6G3-H9 0.990 2.618 155.56 3.543 O6G2

C7G3-H18 0.980 2.425 122.92 3.070 O4G4

C8G3-H8G7 0.980 2.505 140.82 3.323 O2G2

C8G3-H8G7 0.980 2.644 112.63 3.154 O4G3

C8G4-H842 0.980 2.546 115.83 3.101 O2G4

C1G5-H1G5 1.000 2.436 136.09 3.232 O6G6

C2G5-H2G5 1.000 2.356 156.32 3.297 O6G1 [ -x+3/2, -y+1, z-1/2 ]

C6G5-H4 0.990 2.387 127.69 3.094 O5G4

C6G5-H4 0.990 2.516 158.10 3.455 O6G4

C9G5-H29 0.980 2.572 125.05 3.236 O3G1 [ -x+3/2, -y+1, z-1/2 ]

C1G6-H1G6 1.000 2.596 117.41 3.182 O3G7

C6G6-H2 0.990 2.348 135.59 3.133 O5G5

C8G6-H8GX 0.980 2.479 117.79 3.061 O4G6

C9G6-H31 0.980 2.466 117.92 3.050 O3G4 [ x-1, y, z ]

C9G6-H31 0.980 2.549 112.81 3.065 O5G6

C2G7-H2G7 1.000 2.520 154.82 3.452 O6G3 [ x-1, y, z ]

C8G7-H7 0.980 2.479 120.64 3.096 O2G7

**TABLE S4. Geometrical parameters^**^ for the host molecule in the crystal**

**of complex 2 (TMB⋅RALA)**

| **Residue** | ***r*  (Å)** | ***D* (Å)** | ***a* (°)** | ***d* (°)** | ***α*^a^ (Å)** | ***D_3_*^b^ (Å)** | ***τ^c^* (°)** |
| --- | --- | --- | --- | --- | --- | --- | --- |
| G1 | 4.793 | 4.547 | 131.2 | 17.5 | 0.420 | 3.142 | 28.4 |
| G2 | 5.227 | 4.223 | 124.0 | -8.5 | 0.241 | 3.623 | 20.4 |
| G3 | 5.217 | 4.527 | 121.0 | -23.0 | -0.516 | 3.702 | -9.6 |
| G4 | 4.599 | 4.310 | 139.1 | 27.0 | -0.057 | 3.402 | 49.3 |
| G5 | 5.083 | 4.430 | 122.4 | 0.0 | 0.625 | 3.854 | 38.3 |
| G6 | 5.267 | 4.360 | 121.2 | -22.7 | -0.345 | 3.463 | -13.0 |
| G7 | 4.838 | 4.284 | 132.0 | 5.3 | -0.370 | 3.208 | 34.8 |

Mean e.s.d.s: **^a^**0.002 Å; **^b^**0.004 Å; **^c^**0.1°

^**^The listed parameters are defined as follows:

***r***, the distance of each O4 atom from the centroid of the O4-polygon;

***D***, the glycosidic O4⋅⋅⋅O4′ distance;

***a***, the O4⋅⋅⋅O4′⋅⋅⋅O4′′ angle;

***d***, the O4⋅⋅⋅O4′⋅⋅⋅O4′′⋅⋅⋅O4′′′ torsion angle;

***φ***, the intersaccharidic angle C1(n+1)-O4(n)-C4(n);

***D_3_***, the O2⋅⋅⋅O3′ intra-ring distance;

***α***, the deviation of each O4 atom from the mean O4-plane;

***τ***_,_ tilt angle: the angle between the plane containing the atoms O4, C4, C1 and O4′ of a given glucose ring and the mean O4-plane.

**FIGURE S2. Calculated PXRD pattern for complex 2 (TMB⋅RALA)**

(PXRD pattern based on refined single crystal X-ray data at 173(2) K and computed

with program Lazy Pulverix [1] for CuKα_1_ radiation)


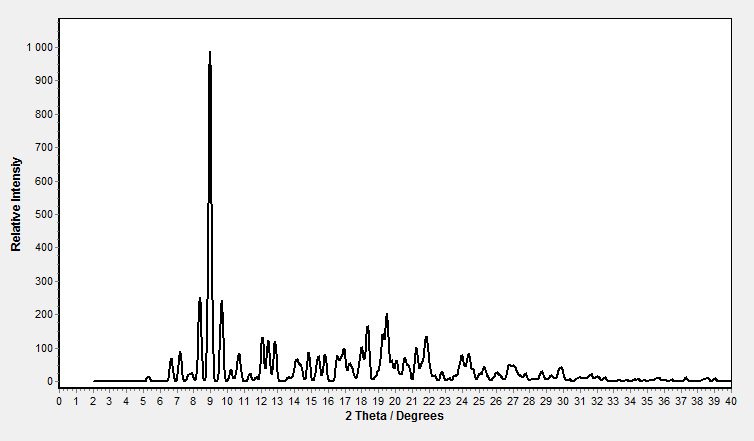


**Relative intensity**

[1]. K. Yvon, W. Jeitschko and E. J. Parthé, *J. Appl. Crystallogr.,* 1977, **10**, 73– 74.
